# Complex CatSper-dependent and independent [Ca^2+^]_i_ signalling in human spermatozoa induced by follicular fluid

**DOI:** 10.1093/humrep/dex269

**Published:** 2017-08-28

**Authors:** Sean G. Brown, Sarah Costello, Mark C. Kelly, Mythili Ramalingam, Ellen Drew, Stephen J. Publicover, Christopher L.R. Barratt, Sarah Martins Da Silva

**Affiliations:** 1 School of Science, Engineering and Technology, Abertay University, Dundee DD11HG, UK; 2 School of Biosciences, The University of Birmingham, Birmingham B15 2TT, UK; 3 Reproductive and Developmental Biology, School of Medicine, Ninewells Hospital and Medical School, University of Dundee, Dundee DD19SY, UK; 4 Assisted Conception Unit, Ninewells Hospital Dundee, Dundee DD19SY, UK

**Keywords:** follicular fluid, patch clamp electrophysiology, CatSper, potassium channel, spermatozoa

## Abstract

**STUDY QUESTION:**

Does progesterone in human follicular fluid (hFF) activate CatSper and do other components of hFF modulate this effect and/or contribute separately to hFF-induced Ca^2+^ signaling?

**SUMMARY ANSWER:**

hFF potently stimulates CatSper and increases [Ca^2+^]_i_, primarily due to high concentrations of progesterone, however, other components of hFF also contribute to [Ca^2+^]_i_ signaling, including modulation of CatSper channel activity and inhibition of [Ca^2+^]_i_ oscillations.

**WHAT IS KNOWN ALREADY:**

CatSper, the principal Ca^2+^ channel in spermatozoa, is progesterone-sensitive and essential for fertility. Both hFF and progesterone, which is present in hFF, influence sperm function and increase their [Ca^2+^]_i_.

**STUDY DESIGN, SIZE, DURATION:**

This basic medical research study used semen samples from >40 donors and hFF from >50 patients who were undergoing surgical oocyte retrieval for IVF/ICSI.

**PARTICIPANTS/MATERIALS, SETTING, METHODS:**

Semen donors and patients were recruited in accordance with local ethics approval (13/ES/0091) from the East of Scotland Research Ethics Service REC1. Activities of CatSper and KSper were assessed by patch clamp electrophysiology. Sperm [Ca^2+^]_i_ responses were examined in sperm populations and single cells. Computer-assisted sperm analysis (CASA) parameters and penetration into viscous media were used to assess functional effects.

**MAIN RESULTS AND THE ROLE OF CHANCE:**

hFF and progesterone significantly potentiated CatSper currents. Under quasi-physiological conditions, hFF (up to 50%) failed to alter membrane K^+^ conductance or current reversal potential. hFF and progesterone (at an equivalent concentration) stimulated similar biphasic [Ca^2+^]_i_ signals both in sperm populations and single cells. At a high hFF concentration (10%), the sustained (plateau) component of the [Ca^2+^]_i_ signal was consistently greater than that induced by progesterone alone. In single cell recordings, 1% hFF-induced [Ca^2+^]_i_ oscillations similarly to progesterone but with 10% hFF generation of [Ca^2+^]_i_ oscillations was suppressed. After treatment to ‘strip’ lipid-derived mediators, hFF failed to significantly stimulate CatSper currents but induced small [Ca^2+^]_i_ responses that were greater than those induced by the equivalent concentration of progesterone after stripping. Similar [Ca^2+^]_i_ responses were observed when sperm pretreated with 3 μM progesterone (to desensitize progesterone responses) were stimulated with hFF or stripped hFF. hFF stimulated viscous media penetration and was more effective than the equivalent does of progesterone.

**LARGE SCALE DATA:**

N/A.

**LIMITATIONS, REASONS FOR CAUTION:**

This was an *in vitro* study. Caution must be taken when extrapolating these results *in vivo*.

**WIDER IMPLICATIONS OF THE FINDINGS:**

This study directly demonstrates that hFF activates CatSper and establishes that the biologically important effects of hFF reflect, at least in part, action on this channel, primarily via progesterone. However, these experiments also demonstrate that other components of hFF both contribute to the [Ca^2+^]_i_ signal and modulate the activation of CatSper. Simple *in vitro* experiments performed out of the context of the complex *in vivo* environment need to be interpreted with caution.

**STUDY FUNDING/COMPETING INTEREST(S):**

Funding was provided by MRC (MR/K013343/1, MR/012492/1) (S.G.B., S.J.P., C.L.R.B.) and University of Abertay (sabbatical for S.G.B.). Additional funding was provided by TENOVUS SCOTLAND (S.M.D.S.), Chief Scientist Office/NHS Research Scotland (S.M.D.S). C.L.R.B. is EIC of MHR and Chair of the WHO ESG on Diagnosis of Male infertility. The remaining authors have no conlicts of interest.

## Introduction

Human follicular fluid (hFF) affects various important functions of human spermatozoa, including hyperactivated motility, chemotaxis and acrosome reaction (Baldi *et al.*, 1998). Almost 30 years ago Thomas *et al.* demonstrated that hFF stimulated a rapid influx of Ca^2+ ^in human spermatozoa (Thomas and Meizel, 1988). Subsequently, progesterone (P4) was shown to have effects on sperm function similar to those of hFF and was found to be the component of hFF that was primarily responsible for induction of Ca^2+^-influx (Osman *et al.*, 1989; Thomas and Meizel, 1989). In 2011, Lishko and Strunker independently showed that induction of Ca^2+^ influx by P4 was via the sperm-specific channel CatSper (Lishko *et al.*, 2011; Strunker *et al.*, 2011), which is now known to be stimulated by a wide range of small organic molecules (Brenker *et al.*, 2012). P4, at high concentrations (~μM range), also inhibits KSper channels (Mannowetz *et al.*, 2013). It has been proposed that high concentrations of P4 encountered in the vicinity of the oocyte and its vestments achieve full activation of CatSper through a combination of CatSper activation and depolarization of membrane potential due to KSper inhibition (Mannowetz *et al.*, 2013).

As P4 is a primary component of hFF, a logical assumption is that exposure of human spermatozoa to hFF *in vivo* activates CatSper. However, the ‘clean’ stimuli that are used for *in vitro* investigations, such as those by which the action of P4 on CatSper was established, differ greatly from the complex environment of the reproductive tract (Mortimer *et al.* 2013; Sakkas *et al.*, 2015). hFF is a complex fluid (Revelli *et al.*, 2009; O'Gorman *et al.*, 2013) and, in its presence, sperm are simultaneously exposed to multiple ligands, potentially leading to multiple separate effects and/or interactions. Significantly, pre-treatment with oestrogen (17βE_2_), which elevates [Ca^2+^]_i_ in spermatozoa apparently by a mechanism independent of CatSper (Luconi *et al.*, 1999; Lishko *et al.*, 2011; Mannowetz *et al.*, 2017), reduced the Ca^2+^ response to subsequent stimulation with P4 (Luconi *et al.*, 1999). Consequently, two fundamental questions are (i) Does hFF act on CatSper in a manner consistent with the previously described effects of its principal component P4, or are there synergistic or even antagonistic effects on CatSper upon exposure to these complex mixtures? (ii) Do other components of hFF contribute significantly, but separately, to hFF-induced Ca^2+^ signalling?

## Materials and Methods

### Experimental solutions

Details for HEPES buffered saline, bicarbonate buffered capacitating medium, supplemented Earle's balanced salt solution (sEBSS), standard bath solution (patch seals and quasi-physiological recording), standard pipette solution (quasi-physiological recording), Cs^+^-based pipette and bath solutions (monovalent CatSper currents) and bath (Ba^2+^) and pipette solutions for CatSper tail currents are given in Supplementary File S1.

### Selection and preparation of spermatozoa

Semen samples were from donors with normal sperm concentration and motility (WHO 2010). Samples were obtained by masturbation after 2–3 days sexual abstinence. After liquefaction, sperm were isolated by either swim-up or density gradient centrifugation (electrophysiological studies) and left to capacitate (37°C, 6% CO_2_) for 3–5 h (Alasmari *et al.*, 2013a). Samples were obtained and analysed in line with suggested guidance for human semen studies and variations identified (Bjorndahl *et al.*, 2016).

### Human follicular fluid

Oocytes were retrieved by transvaginal aspiration 36 h after injection of r-hCG. Most (90%) of these oocytes were in metaphase II. hFF without blood contamination from the largest follicles of each ovary was centrifuged at 2500 g for 10 min to separate cellular components and the supernatant (0.22 μm filtered) was either used on the day for experimentation or stored (at −20°C) until use (<1 week). hFF progesterone (in whole and dextran-coated charcoal-stripped samples) was assayed before use (Siemens ADVIA Centaur®XP competitive Immunoassay System).

### Stripping of steroids, prostaglandins and other lipid-derived components from hFF

Steroids and prostaglandins were removed from hFF by adapting the dextran-coated activated charcoal method for removal of steroids from serum (product information sheet C9157; Sigma-Aldrich, UK; Supplementary File S1).

### Electrophysiology

Currents were recorded from sperm isolated by density gradient using whole-cell patch clamp (Mansell *et al.*, 2014). To investigate K^+^ channel function, cells were studied under quasi-physiological conditions (standard pipette and bath solutions) using a ramp protocol (−92 to 68 mV over 2500 ms). Membrane potential was held at −92 mV between ramps (Brown *et al.*, 2016). Reversal potentials (E_rev_—to estimate resting Vm) and membrane conductance (Gm) were calculated as previously described (Brown *et al.*, 2016). Monovalent CatSper currents were recorded using Cs^+^-based divalent-free pipette and bath solutions. Currents were evoked by a ramp protocol (−80 to 80 mV over 1 s). Membrane potential was held at 0 mV between ramps. Divalent (Ba^2+^) CatSper tail currents (Lishko *et al.*, 2011) were evoked by 400 ms pulses followed by stepping to −150 mV (200 ms). Vm was held at −70 mV between sweeps (Lishko *et al.*, 2011). Tail current amplitudes were used to plot voltage activation (G–V) curves. Data were sampled at 2 kHz, filtered at 1 kHz. Tail current data were leak subtracted using pClamp P/4 protocol to minimize the impact of membrane resistance (PClamp 10 software, Axon instruments).

### Assessment of [Ca^2+^]_i_ signals

#### Population recordings

Following swim-up, sperm (≈6 million/ml) were capacitated (3–5 h) then loaded with 4.5 μM Fluo-4 for 30 min, washed twice (700 g for 10 min) and resuspended in sEBSS. [Ca^2+^]_i_ was assessed using a FLUOstar microplate reader (BMG Labtech Offenburg, Germany) with 488 nm (excitation) and 520 nm (emission) filters. After a control period, (30–60 s) stimuli were added using a multichannel pipetter as described by Strunker *et al.* (2011). To compare [Ca^2+^]_i_ responses to hFF and equivalent [P4] aliquots from the same fluo-4 loaded sample, tests were performed in parallel. Emission was background-corrected and normalized to the control (pre-stimulus) amplitude. To compare duration of P4 and hFF-induced transients, the half-duration (midpoint of the rising phase to midpoint of decay) was calculated. In desensitization experiments, cells were first stimulation with 3 μM P4 then, after a delay of 300 s, a second ‘test’ stimulus was applied in the continued presence of the desensitizing P4.

#### Single cell recordings

Recordings were made as described previously (Nash *et al.*, 2010) but using Fluo-4. All experiments were performed at 25 ± 0.5°C in a continuous flow of medium. Images were captured at 0.2 Hz using a 40× oil objective and Andor Ixon 897EMCCD camera controlled by iQ software (Andor Technology, Belfast, UK). Fluorescence from the sperm posterior head/neck was background-corrected and normalized to give % change in intensity (Nash *et al.*, 2010).

To assess [Ca^2+^]_i_ oscillations, paired experiments were conducted using cells from the same sample exposed to hFF or P4. Traces were examined by eye for the occurrence of cyclical [Ca^2+^]_i_ oscillations following the initial [Ca^2+^]_i_ transient.

### Assessment of sperm function

Viscous media penetration test and Computer-assisted sperm analysis (CASA) were carried out as previously described (Alasmari *et al.*, 2013a; Williams *et al.*, 2015).

### Ethical approval

Written consent was obtained from each IVF patient in accordance with the Human Fertilization and Embryology Authority (HFEA) Code of Practice (V8) under local ethics approval (13/ES/0091) from the East of Scotland Research Ethics Service REC1. Similarly, volunteer sperm donors were recruited under the same ethical approval in Dundee and ethical approval number ERN-12-0570R at the University of Birmingham.

### Data analysis

Data were analyzed using Microsoft Excel™ or GraphPad Prism™ (version 5, GraphPad Software Inc.). Statistical significance was determined using Student's paired/unpaired *t*-test or analysis of variance (ANOVA) and adjusted using the Holm–Bonferroni correction (Gaetano, 2013) as appropriate. Percentage data were ArcSine converted before testing. Data are presented as mean ± SEM with *P* < 0.05 indicative of statistical significance. All sets of experimental repeats include sperm and hFF samples from more than one donor. Values of ‘*n*’ for patch clamp experiments are given in Tables I–VI and show the number of cells patched. Unless stated otherwise, the values of ‘*n*’ for [Ca^2+^]_i_ and motility assessments provided in text and figure legends show the number of experiments used for statistical analysis.

**Table I:** Effect of hFF on monovalent (Cs^+^) CatSper current amplitude.

		−80 mV			80 mV		
Stimulus	*n*	Control (pA)	Treated (pA)	*P*	Control (pA)	Treated (pA)	*P*
1%hFF	13	−89.4 ± 8.3	−199 ± 33.6	0.01	193.3 ± 18.4	507.3 ± 37.7	0.001

**Table II:** Effect of hFF on CatSper V_50_.

Stimulus	*n*	Control (mV)	Treated (mV)	*P*
1% hFF	12	61.8 ± 5.2	25.1 ± 2.7	<0.001
500 nM P4	4	71.7 ± 8.0	15.1 ± 6.1	<0.01

**Table III:** Effect of stripped hFF (ShFF) on monovalent (Cs^+^) CatSper current amplitude.

		−80 mV			80 mV		
Stimulus	*n*	Control (pA)	Treated (pA)	*P*	Control (pA)	Treated (pA)	*P*
1%ShFF	8	−130.3 ± 28.9	−105.6 ± 32.2	0.013	300.8 ± 68.6	258.7 ± 74.9	0.07
1%hFF	8	−130.3 ± 28.9	−189.9 ± 52.0	0.05	300.8 ± 68.6	431.5 ± 85.8	0.008

**Table IV:** Effect of stripped hFF (ShFF) on CatSper V_50_.

Stimulus	*n*	Control (mV)	Treated (mV)	*P*
1% ShFF	4	54.0 ± 10.8	51.0 ± 8.8	NS
1% hFF	4	54.0 ± 10.8	9.3 ± 4.0	0.01

**Table V:** Is failure of 1% ShFF to potentiate CatSper currents due to contamination with divalent cations?

		−80 mV			80 mV		
Stimulus	*n*	Control (pA)	Treated (pA)	*P*	Control (pA)	Treated (pA)	*P*
2 nM P4	4	−60.3 ± 13.5	−90.0 ± 18.9	0.02	193.4 ± 23.7	237.4 ± 36.7	0.046
2 nM P4 with Ca/Mg	5	−62.1 ± 16.7	−111.9 ± 21.7	0.002	156.6 ± 22.1	213.2 ± 16.0	0.012
ShFF with 9 mM EGTA, 9 mM EDTA	17	−98.9 ± 14.4	−125.6 ± 21.7	0.12	214.6 ± 24.7	223.9 ± 31.7	0.62

**Table VI:** Effect of hFF on K^+^ current reversal potential and conductance.

		Erev (mV)			Gm (ns/pF)		
Stimumlus	*n*	Control (pA)	Treated (pA)	*P*	Control (pA)	Treated (pA)	*P*
1% hFF	6	−34.6 ± 4.4	−36.5 ± 6.6	>0.05	1.02 ± 0.17	1.12 ± 0.21	>0.05
10% hFF	3	−22.0 ± 9.0	−22.8 ± 9.1	>0.05	0.79 ± 0.20	0.72 ± 0.25	>0.05
50% hFF	3	−23.95 ± 3.8	−24.0 ± 4.0	>0.05	0.64 ± 0.06	0.57 ± 0.04	>0.05
10 μM P4	3	−28.2 ± 2.8	−18.28 ± 4.6	0.09	0.51 ± 0.06	0.41 ± 0.03	0.32
30 μM P4	4	−41.4 ± 3.5	−21.0 ± 5.5	0.023	0.68 ± 0.08	0.25 ± 0.06	0.026

## Results

### hFF and ion channel currents

#### Effects of hFF on CatSper current

**Figure 1:**
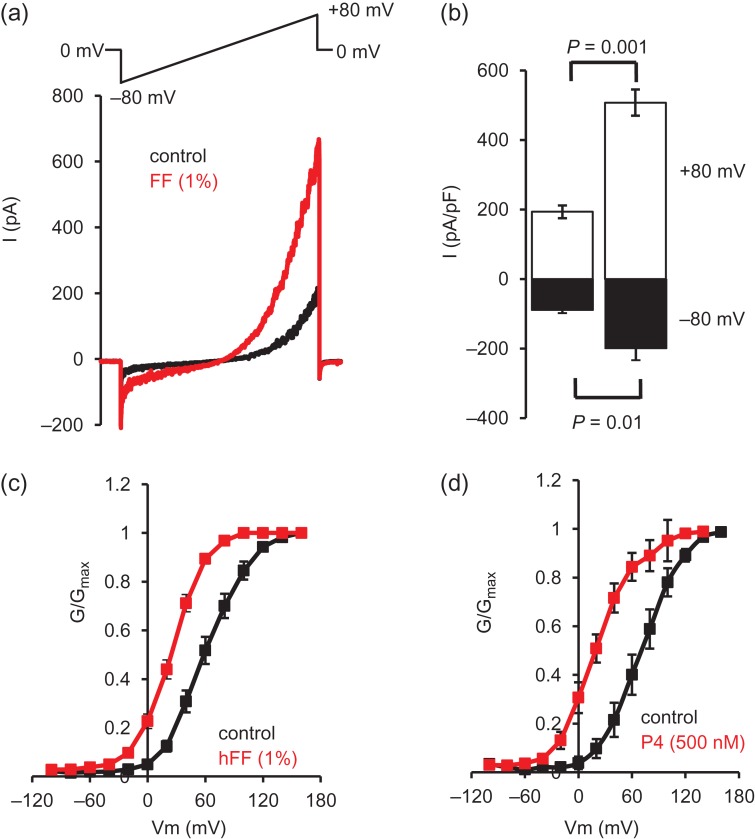
hFF potentiates CatSper currents and shifts the voltage sensitivity to less depolarized potentials. (**a**) Representative Cs^+^-mediated CatSper current in the absence (black) and presence (red) of 1% hFF. Voltage protocol imposed is shown above. (**b**) Mean amplitudes (±SEM) of CatSper currents recorded in the absence (left) and presence (right) of 1% hFF (*n* = 8 hFF samples). Black bars show inward current (−80 mV), white bars show outward currents (80 mV; *n* = 13). (**c** and **d**) Show conductance-voltage (G–V) relationships for Ba^2+^-mediated CatSper tail currents in the absence and presence of 1% hFF (c, *n* = 12) and 500 nM P4 (d, *n* = 4). hFF, human follicular fluid.

Since P4 is an activator of CatSper, we first used whole-cell patch clamp electrophysiology to examine the effect of hFF on CatSper currents (I_CatSper_). hFF (diluted 1%) potently potentiated both inward and outward monovalent CatSper currents (Fig. 1a,b; Table I; *P* < 0.01). P4 potentiates CatSper currents primarily by shifting channel activation to more negative voltages (Lishko *et al.*, 2011). Assessment of voltage sensitivity of CatSper activation (using Ba^2+^ tail currents) showed that 1% hFF shifted the G–V curve to more negative voltages (Fig. 1c), significantly changing the V_50_ (Table II; *P* < 0.001). Similarly, 500 nM P4 caused a negative shift of the CatSper G–V curve (Fig. 1d, Table II; *P* < 0.01) as demonstrated previously (Lishko *et al.*, 2011).

#### Effects of steroid stripping on hFF-stimulation of CatSper currents

**Figure 2:**
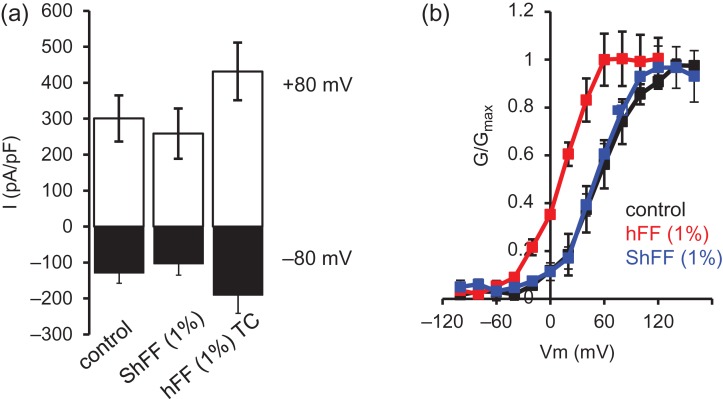
Charcoal-stripped hFF (ShFF) does not potentiate CatSper currents. (**a**) Mean ± SEM inward CatSper currents at −80 mV (black) and outward currents at 80 mV (white; *n* = 8 cells) under control conditions, in presence of 1% stripped hFF (ShFF) and 1% time-control (hFF; 7FF samples). ShFF reduced current amplitude (*P* < 0.05) but subsequent application of control hFF potentiated both inward and outward currents (*P* < 0.01 compared to ShFF). (**b**) 1% stripped hFF (ShFF) failed to alter CatSper voltage sensitivity but subsequent application of control follicular fluid (hFF) caused a significant leftward shift in voltage sensitivity (V_50_*P* < 0.01 compared to control and ShFF). *n* = 4 cells, four hFF.

hFF contains, in addition to P4, prostaglandins (Lishko *et al.*, 2011) and other ligands that may influence [Ca^2+^]_i_ signalling. To examine the effect of depleting lipid-derived agonists (steroids and prostaglandins), samples of FF were ‘stripped’ using dextran-coated charcoal. This procedure reduced [P4] by 98.6 ± 0.13% (*n* = 31; Supplementary Fig. S1). Spermatozoa were exposed first to 1% charcoal-stripped hFF (ShFF) then to 1% hFF from the same sample incubated similarly but without dextran-coated charcoal. ShFF failed to stimulate I_CatSper_, (both inward and outward currents were smaller; Fig. 2a; Table III; *P* < 0.05), but subsequent application of hFF potentiated both inward and outward currents amplitude (Fig. 2a; Table III; *P* = 0.05; *P* < 0.01, respectively). Similarly, when tail currents were used to assess CatSper activation, hFF but not ShFF shifted voltage sensitivity to less positive potentials (Fig. 2b; Table IV; *P* < 0.01). The concentration of P4 present in 1% ShFF is 2–3 nM, which has been reported to increase CatSper currents (Lishko *et al.*, 2011). We therefore assessed whether we could detect this effect under our recording conditions. Both using standard Cs^+^ saline recording (P4 added directly to Cs^+^ saline before perfusion of the recording chamber) and also when progesterone was first dissolved in a mixture of 1% standard bath solution (containing 2 mM Ca^2+^, 0.7 mM Mg^2+^) and 99% Cs^+^ saline (to mimic ionic conditions in ShFF experiments), superfusion of sperm with 2 nM P4 significantly increased both outward and inward currents (Table V). Finally, we increased the concentrations of divalent chelators (EGTA, EDTA) in our Cs^+^ recording saline to 9 mM of each to chelate any residual Ca^2+ ^and Mg^2+^ from the hFF. Under these conditions, we observed a response to ShFF in some cells (Supplementary Fig. S2) and mean inward and outward currents were increased, but this effect was not significant (Table V; *P* > 0.1). Examination of [P4] concentrations showed that detectable effects of ShFF occurred only with hFF samples where the [P4] was unusually high (Supplementary Fig. S2).

#### Effect of hFF on membrane potential and K^+^ current

**Figure 3:**
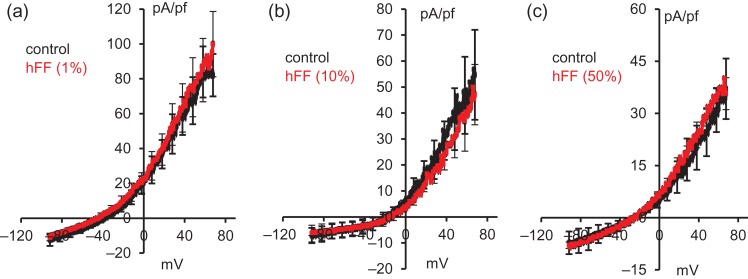
hFF does not affect K^+^ channel activity recorded under quasi-physiological conditions. In each panel, black trace shows mean (±SEM) control current and red trace shows mean (±SEM) of currents recorded after exposure to hFF. (**a**) 1% hFF; *n* = 6 cells, four hFF tested; (**b**) 10% hFF; *n* = 3 cells, three hFF tested; (**c**) 50% hFF; *n* = 3 cells, three hFF.

To investigate the possible effects of hFF on membrane potential, cells were challenged with hFF (1, 10 and 50% dilution) under quasi-physiological conditions (see Materials and Methods section). hFF did not alter resting membrane potential or outward membrane conductance indicating that hFF did not modulate/suppress K^+^ channel function at these dilutions (Fig. 3; Table VI). Stimulation with P4 significantly depolarized membrane potential and reduced conductance at 30 μM but at 10 μM effects were not significant (Table VI).

### hFF and sperm [Ca^2+^]_i_

#### hFF-induced [Ca^2+^]_i_ signals in sperm populations

**Figure 4:**
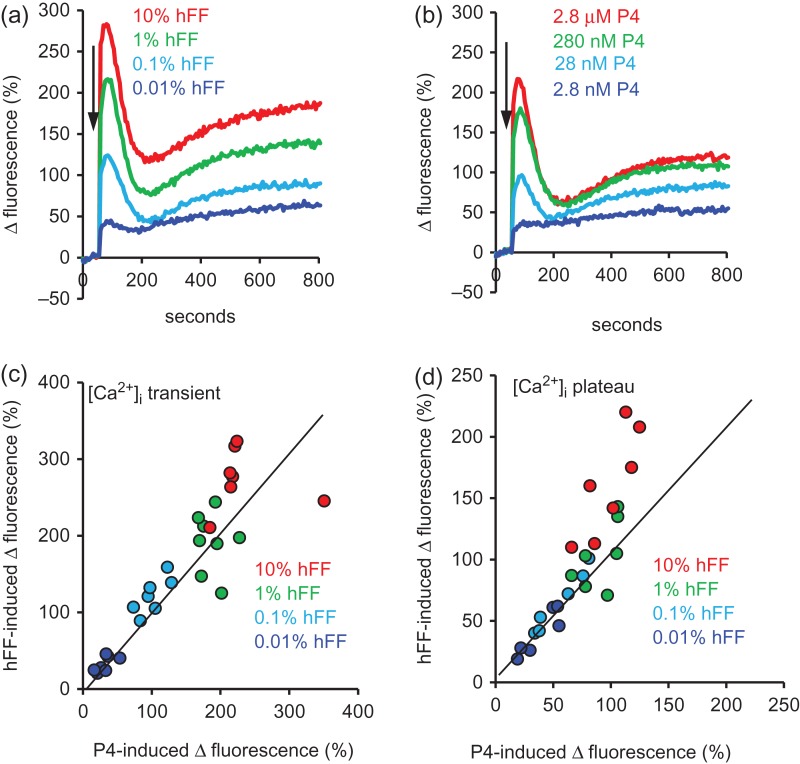
[Ca^2+^]_i_ responses to hFF and progesterone are similar but not identical. (**a** and **b**) Show an example of [Ca^2+^]_i_ responses induced in paired experiments using (a) four dilutions of hFF (dark blue = 0.01%, light blue = 0.1%, green = 1%, red = 10%) and (b) P4 at concentrations equivalent to those in the hFF dilutions (dark blue = 2.8 nM, light blue = 28 nM, green = 280 nM, red = 2.8 μM). (**c** and **d**) Show relative amplitudes (Δ fluorescence (%)) of the [Ca^2+^]_i_ transients (c) and [Ca^2+^]_i_ plateau (d, assessed 10 min post-stimulation) induced in seven sets of experiments, each using four dilutions of hFF (0.01% = dark blue, 0.1% = light blue, 1% = green, 10% = red) and P4 at concentrations equivalent to those in the hFF dilutions. Six different hFF samples were used. Line in each graph marks position of equal response amplitude. At the highest hFF concentration used (10%; red symbols), plateau responses are consistently larger than those of equivalent [P4] (*P* = 0.001).

In agreement with previous reports hFF, similarly to P4, caused a dose-dependent, biphasic elevation of [Ca^2+^]_i_ consisting of a transient followed by a plateau (Fig. 4a,b). Using hFF samples in which the P4 concentration had been determined we directly compared [Ca^2^^+^]_i_ signals induced by hFF (diluted to 10, 1, 0.1 and 0.01%) and by an equivalent concentration of P4 alone (using aliquots of sperm from the same batch of Fluo-4 loaded sperm cells run in parallel). Analysis of these data pairs showed that at low concentrations of hFF (0.01–1%) the amplitudes of signals induced by hFF and P4 were similar (Fig. 4c,d). However, at the highest hFF concentration (10%) the [Ca^2+^]_i_ plateau induced by hFF (assessed 10 min after stimulus application) was consistently greater than that induced by an equivalent concentration of P4 (mean amplitude ratio hFF:P4 = 1.6 ± 0.1; Fig. 4d red symbols; *P* = 0.001; *n* = 7;). In cells stimulated with 10% hFF the [Ca^2+^]_i_ transient also appeared longer than in cells from the same batch of Fluo-4 loaded sperm cells stimulated with an equivalent concentration of P4 (Fig. 4a,b). Assessment of the transient ‘half-duration’ (latency from midpoint of the rising phase to midpoint of decay) confirmed that this was the case (*P* = 0.0005; *n* = 7).

#### hFF-induced [Ca^2+^]_i_ signals in single cells

**Figure 5:**
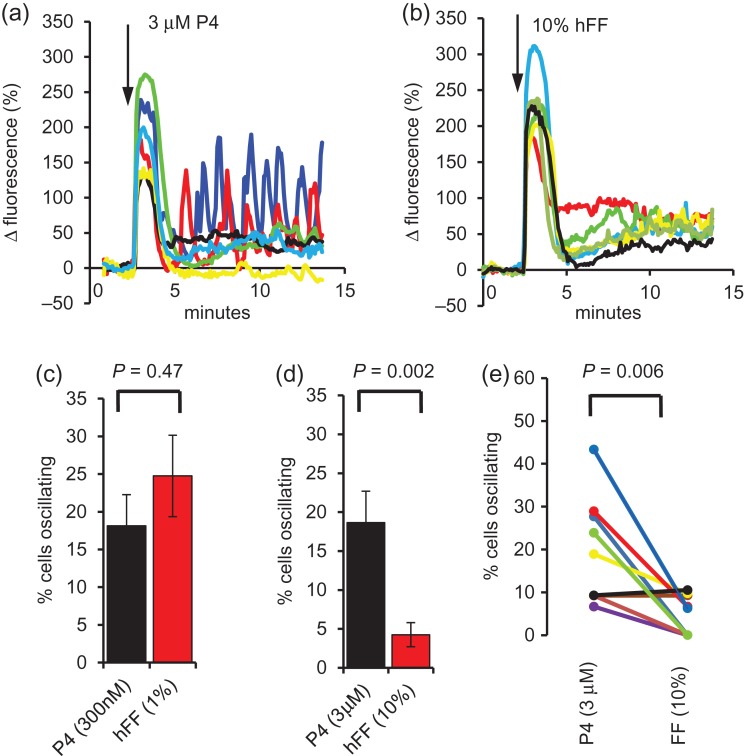
Single cell [Ca^2+^]_i_ responses to hFF. (**a** and **b**) Show examples of [Ca^2+^]_i_ responses in a paired experiment in which cells from the same sample were exposed to 3 μM P4 (a) and 10% hFF (b). Panel **c** shows mean ± SEM percentage of cells in which [Ca^2+^]_i_ oscillations occured after stimulation of sperm (from the same sample) with 300 nM P4 (black) or 1% hFF (red); *n* = 10 paired experiments. Panel **d** shows results from a similar series of 10 paired assessments using 3 μM P4 (black) and 10% hFF (red; *P* < 0.01). (**e**) Shows data from the 3 μM P4/10% hFF experiments summarized in panel d with paired experiments joined and shown in same colour.

Similarly to population measurements, single cell imaging of [Ca^2+^]_i_ at the posterior head/neck showed transient responses in the vast majority of cells exposed to hFF, which resembled those induced by P4 alone (Fig. 5a,b). In P4-stimulated cells the initial Ca^2+^ transient was often followed by [Ca^2+^]_i_ oscillations (not synchronized and therefore detectable only in single cell records; Harper *et al.*, 2004; Kirkman-Brown *et al.*, 2004; Fig. 5a). In cells stimulated with hFF, oscillations were observed but their occurrence was markedly concentration dependent. 1% hFF, similarly to 300 nM P4 (estimated equivalent [P4]) induced oscillations in ≈25% of cells (Fig. 5c; *P* = 0.47; *n* = 10). However, whereas 3 μM P4 was similarly effective (19% of cells; e.g. Fig. 5a), 10% hFF-induced oscillations in only 4% of cells (Fig. 5b,d,e; *P* = 0.002, *n* = 10).

#### [Ca^2+^]_i_ responses to charcoal-stripped hFF

**Figure 6:**
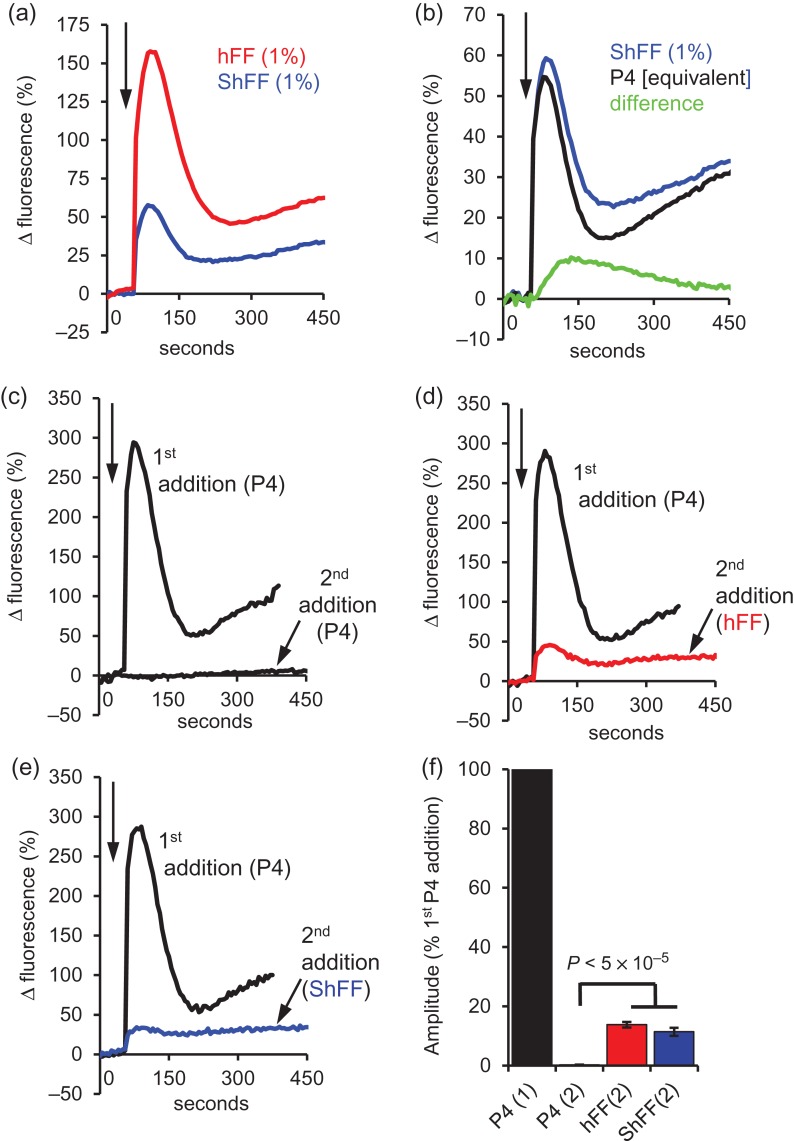
Components of the hFF-induced [Ca^2+^]_i_ signal are resistant to P4 desensitization and charcoal stripping. (**a**) Mean [Ca^2+^]_i_ response from 21 experiments (5 different hFF used) in which aliquots from the same sperm sample treated with 1% hFF (red) and 1% ShFF (blue). (**b**) Mean [Ca^2+^]_i_ response from 28 paired experiments (9 different hFF used) in which aliquots from the same sperm sample were treated with 1% ShFF (blue) or the equivalent concentration of P4 (black). Green shows the ‘non-P4’ component obtained by subtraction of traces. (**c**–**e**) Examples of [Ca^2+^]_i_ responses in three parallel recordings where sperm were first stimulated with 3 μM P4 (first addition-black traces) then, after an interval of 5 min, exposed to either a second 3 μM P4 stimulus (6 μM P4 total; c, second addition-black trace), 1% hFF (d, second addition-red trace) or 1% ShFF (e, second addition-blue trace). In each panel the responses to the first (3 μM P4) stimulus and to the second stimulus are overlaid (arrow at top left shows time of additions). When 3 μM P4 was followed by a second P4 stimulus the second response was negligible (desensitization). However, when either 1% hFF or 1% ShFF was added as the second stimulus there was a small transient followed by a plateau. (**f**) Mean amplitude (±SEM) of [Ca^2+^]_i_ transients evoked by the first 3 μM P4 stimulus (P4(1) black) and by a second addition of P4 (P4(2); *n* = 7; black), hFF (hFF(2); *n* = 10; red) or stripped hFF (ShFF(2); *n* = 6; blue). All amplitudes are normalized to that induced by the first P4 addition in that experiment.

Since the ability of 1% hFF to potentiate CatSper currents was removed by stripping of steroids/prostaglandins with dextran-treated charcoal (Fig. 2a), we examined whether hFF-induced [Ca^2+^]_i_ signals were similarly affected. Surprisingly, [Ca^2+^]_i_ responses were always detected in cell populations stimulated with 1% ShFF, with the [Ca^2+^]_i_ transient amplitude being 36.8 ± 1.8% of that in the parallel control (1% hFF) experiments (Fig. 6a; Supplementary Fig. S3; *P* = 3.2 × 10^−12^; *n* = 21). In 28 experiments where parallel recordings were carried out with ShFF and [P4] equivalent to that in ShFF, [Ca^2+^]_i_ transient amplitudes were similar (*P* = 0.14). However, the subsequent [Ca^2+^]_i_ ‘plateau’ was significantly greater with ShFF (43 ± 9% for the period 30-240 s post-stimulus; *P* = 4.8 × 10^−6^; Fig. 6b). The ‘non-P4’ component, isolated by subtraction of traces (ShFF-equivalent [P4]), showed activation later than the [Ca^2+^]_i_ signal induced by P4 and peaked 60–100 s after stimulation (Fig. 6b).

**Figure 7:**
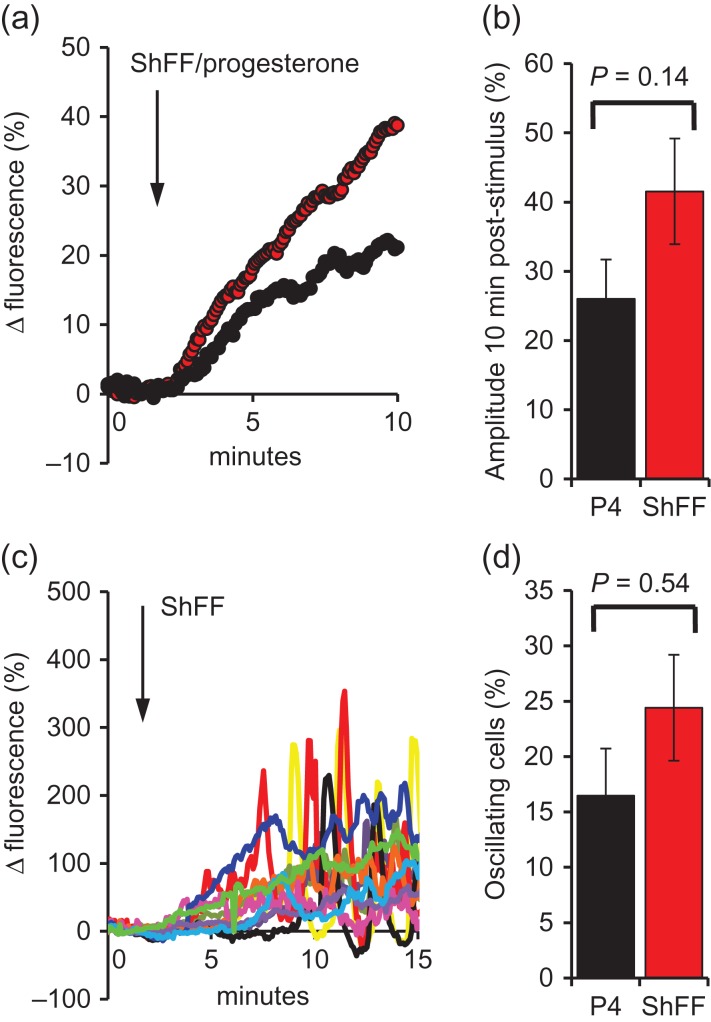
Single cell [Ca^2+^]_i_ responses to 1% ShFF. (**a**) Shows mean responses to 1% ShFF (red; *n* = 10 experiments; 826 cells) and equivalent [P4] (black; *n* = 6 experiments; 447 cells), arrow marks stimulus addition. Both stimuli induced a [Ca^2+^]_i_ ramp rather than the biphasic response seen in fluorimetric experiments. (**b**) Shows mean (±SEM) amplitude (Δ fluorescence) 9 min after stimulus application. (**c**) Shows responses of 12 individual cells stimulated with ShFF, arrow marks stimulus addition. Red, yellow and black cells developed oscillations 5–10 min after stimulation. (**d**) shows proportions of cells generating [Ca^2+^]_i_ oscillations after stimulation with 1% ShFF (red; *n* = 10 experiments; 826 cells) or equivalent [P4] (black; *n* = 6 experiments; 447 cells).

In single cell imaging experiments where immobilized sperm were superfused with 1% ShFF or equivalent [P4], cells failed to generate the [Ca^2+^]_i_ transient seen in the equivalent population experiments and instead we observed a slow [Ca^2+^]_i_ ramp (Fig. 7 a). This reduced efficacy of stimuli delivered by perfusion is due to binding of progesterone to the perfusion tubing (see Discussion section). The mean increase in [Ca^2+^]_i_ was greater in the ShFF-treated cells, but the effect was highly variable and the difference was not significant (Fig. 7a,b; *P* = 0.14). After 5–10 min exposure to 1% ShFF or equivalent [P4], oscillations developed in ~20% of cells (Fig. 7c,d), resembling the response to P4 ramps (Harper *et al.*, 2004).

### Effects of P4 desensitization on [Ca^2+^]_i_ response to hFF

Component(s) of hFF not removed by charcoal stripping contribute significantly to late/sustained components of hFF-induced [Ca^2+^]_i_ signals (Fig. 6b). To further investigate this, we tested the effect of desensitization of the P4 response on the [Ca^2+^]_i_ signal induced by hFF. As previously described (Aitken *et al.*, 1996; Schaefer *et al.*, 1998), when sperm were pre-stimulated with 3 μM P4 complete desensitization occurred (Fig. 6c). However, when P4-desensitized cells were stimulated with hFF there was a clear response (13.8 ± 0.9% of that evoked by the preceding, desensitizing P4 stimulus; *P* = 3.2 × 10^−5^ compared to second stimulation with 3 μM P4; *n* = 10; Fig. 6d,f). Since P4 and prostaglandins stimulate CatSper by separate mechanisms that do not cross-desensitize (Schaefer *et al.*, 1998), this could reflect a small contribution of prostaglandins to the hFF-induced [Ca^2+^]_i_ transient. We therefore investigated whether the desensitization-resistant component of hFF was removed by charcoal stripping. In six experiments ShFF always induced a [Ca^2+^]_i_ response (11.5 ± 2.0% of that evoked by the desensitizing 3 μM P4 stimulus) which was significantly greater (*P* = 2.8×10^−5^) than the response to a second stimulation with 3 μM P4; Fig. 6e,f.

### hFF and sperm motility

To assess functional effects of hFF on motility, we measured hyperactivation and penetration into viscous medium. Both hFF (1 and 10%) and equivalent [P4] significantly stimulated penetration (*P* < 0.005; *n* = 6) but the effect of hFF was significantly greater (Supplementary Fig. S4). hFF also induced a dose-dependent increase in hyperactivation, whereas the effect of equivalent [P4] was small and not significant (*P* < 0.05; *n* = 6; Supplementary Fig. S5a). Analysis of the kinematics (VCL, ALH, LIN) indicated this effect of hFF was primarily due to increased curvilinear velocity (*P* < 0.01; Supplementary Fig. S5b).

## Discussion

Our findings clearly show that CatSper is activated by hFF and that this is the primary contribution to hFF-induced [Ca^2+^]_i_ signalling in human sperm. However, by direct comparison of responses to hFF and to equivalent [P4], charcoal stripping of hFF and desensitization of the P4 response, we identified clear differences between the responses to hFF and to P4 which indicate that regulation of [Ca^2+^]_i_ by hFF is considerably more complex than simple activation of CatSper.

### Modulation of ion channel activity and [Ca^2+^]_i_ by hFF

The electrophysiological data clearly show that hFF, similarly to P4, enhances CatSper currents and shifts CatSper voltage sensitivity to less positive potentials (Fig. 1; Tables I and II). Mannowetz *et al. *(2013) reported that high concentrations of P4 also inhibit KSper (I_50_ ≈ 7 μM), depolarizing the membrane potential and potentially augmenting activation of CatSper. We could detect no effect of hFF on conductance or resting Vm even with 50% hFF (containing 10–15 μM progesterone; Fig. 3). In positive control experiments with P4, we saw no significant effect with 10 μM but clear inhibition of conductance with 30 μM P4 (equivalent [P4] to 100% hFF; Table VI). Thus effects of hFF on KSper may occur at higher concentrations than those used in this study, potentially in very close proximity to the oocyte.

### [Ca^2+^]_i_ signals induced by hFF

[Ca^2+^]_i_ transients induced by treatment of human sperm suspensions with hFF were similar in amplitude to those induced by an equivalent [P4] and activation of CatSper by P4 is apparently the primary determinant of this response. However, when sperm were stimulated with 10% hFF, the sustained [Ca^2+^]_i_ signal was >60% greater than that induced by an equivalent [P4]. Recently, Mannowetz *et al.* reported that endogenous steroids other than P4 also modulate activity of CatSper in human sperm. 17 beta-estradiol and hydrocortisone, both present in hFF, inhibit the stimulatory action of 1 μM P4 (IC_50_ = 833 and 153 nM, respectively) and their actions might be expected to result in a response to hFF smaller than that of an equivalent [P4] (Mannowetz *et al.* 2017). The concentration of P4 in hFF (typically > 30 μM) may be high enough for these inhibitory effects to be outcompeted (Mannowetz *et al.*, 2017), but the stimulatory effects observed with 10% hFF indicate that other components of hFF, when present at sufficient concentration, either activate (or suppress inactivation of) CatSper or activate other [Ca^2+^]_i_ signalling components that contribute to the sustained [Ca^2+^]_i_ signal (see below).

Single cell [Ca^2+^]_i_ responses to P4 resemble population responses (transient and plateau phase; Kirkman-Brown *et al.*, 2000) but some cells then generate repetitive oscillations (Fig. 5a; Harper *et al.*, 2004; Kirkman-Brown *et al.*, 2004) that may regulate motility and/or acrosome reaction (Harper *et al.*, 2004; Bedu-Addo *et al.*, 2007; Sánchez-Cárdenas *et al.*, 2014; Alasmari *et al.*, 2013a,b). In paired experiments, 1% hFF and 300 nM progesterone (equivalent concentration) both induced repetitive [Ca^2+^]_i_ oscillations in ~20% of cells (Fig. 5c), while 1% ShFF and matched [P4], (after a latency of 5–10 min) were similarly effective. However, when challenged with 10% hFF, just 4% of sperm generated oscillations compared to 19% with 3 μM (equivalent) P4 (Figs 5d,e), again suggesting that substances within hFF modulate human sperm Ca^2+^ signalling by mechanisms other than CatSper activation. Darszon *et al.* assessed [Ca^2+^]_i_ and acrosomal status and concluded that calcium oscillations suppress the acrosome reaction (Sánchez-Cárdenas *et al.*, 2014). If the sperm encounters high concentrations of hFF on approaching the cumulus-oocyte complex, this may inhibit [Ca^2+^]_i_ calcium oscillations and ‘disinhibit’ acrosome reaction.

### Charcoal stripping and evidence for presence of an active ‘cocktail’ in hFF

To further investigate the relative contributions of P4 and other components to the observed effects of hFF, samples were treated with dextran-coated charcoal to ‘strip’ lipid-derived agonists (steroids/prostaglandins), removing almost 99% of P4. In fluorimetric experiments the [Ca^2+^]_i_ transients evoked by ShFF were consistent with a response to the residual P4, but the subsequent sustained [Ca^2+^]_i_ signal was significantly greater (Fig. 6b). Furthermore, when we pretreated sperm with P4 to desensitize the P4-induced [Ca^2+^]_i_ signal (Aitken *et al.*, 1996; Schaefer *et al.*, 1998), we found that a small, sustained response persisted whether stimulating with hFF or ShFF (Fig. 6c–f). These observations indicate that hFF includes factors that contribute to and/or regulate Ca^2+^-signalling that are resistant to stripping with dextran-coated charcoal and are therefore unlikely to be steroids or prostaglandins.

Though the [Ca^2+^]_i_ transient induced by 1% ShFF appeared to be primarily a response to residual P4 (see above), when we investigated effects on patch-clamped sperm we observed no stimulation of CatSper currents, suggesting that other components of hFF modulate the response to P4. Two factors should be taken into account in interpreting these data. First, P4 applied by perfusion binds to the plastic perfusion tubing (as evidenced by reduced efficacy of P4 in our imaging experiments and also observed by others; T Strunker personal communication), thus comparison with fluorimetric [Ca^2+^]_i_ assessment, where direct addition of ShFF to the well induced a significant [Ca^2+^]_i_ response (Fig. 6), is misleading. This is particularly significant since the inhibitory effect of hFF was masked at higher [P4] (Supplementary Fig. S2). Second, divalent cations in hFF (2.2 mM Ca, 0.68 mM Mg; Chong *et al.*, 1977; Ng *et al.*, 1987) may be inadequately buffered, masking any stimulatory effect (IC_50_ for Ca^2+^~100 nM; Lishko *et al.*, 2011). However, (i) in ‘supplemented’ control experiments where Ca^2+^/Mg^2+^ was present at equivalent levels to that in ShFF, responses to 2 nM P4 resembled those seen in ‘divalent-free’ controls (Table V) and (ii) increased divalent cation buffering (calculated [Ca^2+^] + [Mg^2+^] with 1% ShFF = 2.14 nM) failed to rescue stimulation of CatSper currents by ShFF (Table V; Supplementary Fig. S2). We conclude that residual P4 in 1% ShFF (a [P4] sufficient to activate CatSper in divalent cation-supplemented control recordings (Table V)), when delivered by perfusion tubing, failed significantly to potentiate CatSper current and propose that other substances present in hFF, resistant to charcoal stripping, partially inhibit the response of the channel to low (nM) concentrations of P4. Thus, the slowly-developing ShFF-induced [Ca^2+^]_i_ ramp seen in imaging experiments (Fig. 7a,c) is apparently induced independently of CatSper activation. The complexity of hFF, even after charcoal stripping, is such that discussion of the nature of such an effect can only be speculative. However, the effects on human sperm [Ca^2+^]_i_ of kisspeptin (Pinto *et al.*, 2012) and leutenising hormone (López-Torres *et al.*, 2017), suggest that activation G-protein coupled receptors by protein or peptide hormones might exert such an effect.

### Functional effect of hFF

We reported previously that stimulation of penetration into artificial mucus was mediated by activation of CatSper whereas manoeuvres designed to mobilize stored Ca^2+^ strongly stimulate hyperactivation (Alasmari *et al.*, 2013a,b). Analysis of motility showed that hFF potently stimulated penetration into viscous medium and also induced a small but significant increase in hyperactivation. Both these effects exceeded those of equivalent [P4], consistent with the significantly greater effects of hFF on [Ca^2+^]_i_ signalling and the likelihood that hFF recruits stored Ca^2+^ in addition to activation of CatSper. These data suggest that stimulation by hFF may contribute significantly to sperm penetration of the cumulus matrix.

In conclusion, the assumption that hFF stimulates CatSper similarly to P4 is correct but a comparison of responses to hFF and P4, particularly at high hFF concentrations or using charcoal-stripped samples, reveal supplementary and modulatory effects of other, unidentified components of hFF. Thus, the mixtures/fluids that the sperm encounters *in vivo* appear to have subtly different and more complex effects than those observed in single agonist, *in vitro* experiments. To understand modulation of sperm function by the reproductive tract, we will need to study more physiological systems.

## Supplementary data

Supplementary data are available at *Human Reproduction* online.

## Authors' role

S.G.B. performed patch clamp experiments. S.C and M.K. performed fluorimetry/imaging and sperm function experiments. M.R. and S.M.D.S were involved in recruiting patients and seeking informed consent. E.D. processed the follicular fluid samples. S.G.B, and S.J.P. performed analysis of electrophysiological data. S.J.P., S.G.B., M.R, S.M.D.S and C.L.R.B. designed the study. S.J.P., S.G.B and C.L.R.B. obtained funding for the study. The manuscript was drafted by C.L.R.B, S.G.B and S.J.P. All authors contributed to the construction, writing and approval the final manuscript.

## Funding

Funding was provided by Medical Research Council (MRC) (MR/K013343/1, MR/012492/1) (S.G.B., S.J.P., C.L.R.B.) and University of Abertay (sabbatical for S.G.B.). Additional funding was provided by Tenovus Scotland (S.M.D.S.), Chief Scientist Office/NHS Research Scotland (S.M.D.S).

## Conflict of interest

C.L.R.B. is EIC of MHR and Chair of the WHO ESG on Diagnosis of Male infertility. The remaining authors have no conflict of interest.

## Supplementary Material

Supplementary Data

Supplementary Data

Supplementary Data

Supplementary Data

Supplementary Data

Supplementary Data
