# Supplementary material for: Complex CatSper-dependent and independent [Ca2+]i signalling in human spermatozoa induced by follicular fluid
Source: Hum Reprod. 2017 Aug 28;32(10):1995–2006. doi: 10.1093/humrep/dex269 (PMC5850303; doi:10.1093/humrep/dex269)
Supplement: Supplementary Data [file dex269_supplementaryfigures1.pdf]

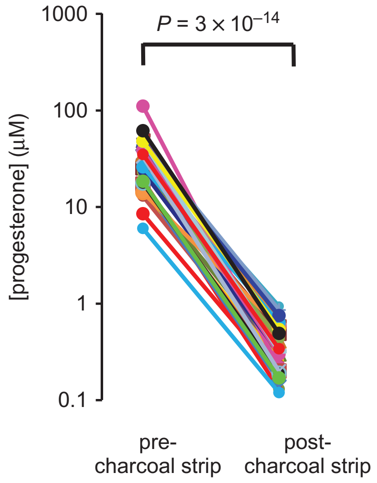

Supplementary Figure S1 Concentrations of P4 in 31 hFF samples assessed before (left) and after (right) stripping of lipid-derived molecules with dextran-coated charcoal. hFF, human follicular fluid.
