# Supplementary material for: Complex CatSper-dependent and independent [Ca2+]i signalling in human spermatozoa induced by follicular fluid
Source: Hum Reprod. 2017 Aug 28;32(10):1995–2006. doi: 10.1093/humrep/dex269 (PMC5850303; doi:10.1093/humrep/dex269)
Supplement: Supplementary Data [file dex269_supplementaryfigures2.pdf]

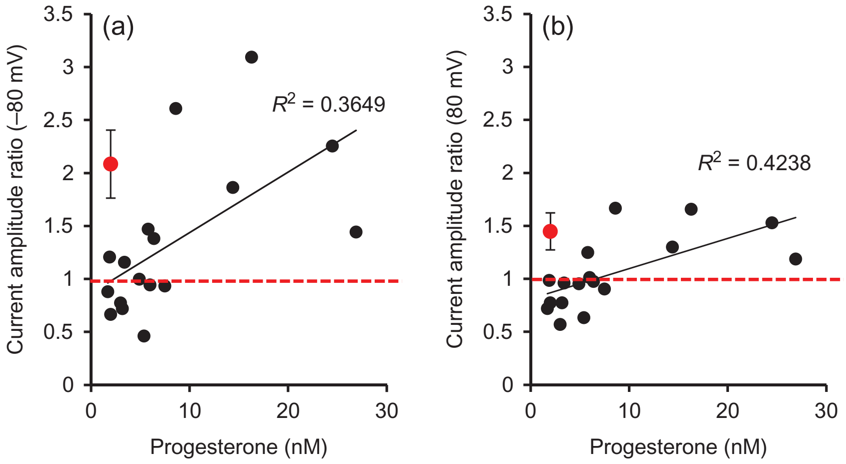

Supplementary Figure S2 Relationship between concentration of progesterone in ShFF and current amplitude ratio (stimulated amplitude: control amplitude). Inward currents (−80 mV) and outward currents (80 mV) are shown in panels a and b show, respectively. Progesterone concentrations shown (17 different hFF used) are after dilution to 1% as used in the experiment. Black symbols show responses of cells treated with 1% ShFF diluted in Cs^+ recording saline containing 9 mM EDTA and 9 mM EGTA, red symbols shows mean ± SEM for five experiments where ShFF was replaced with standard bath solution (containing 2 mM Ca^{2+}, 0.7 mM Mg^{2+} and 200 nM progesterone). Fitted linear regressions are shown on each plot.
