# Supplementary material for: Complex CatSper-dependent and independent [Ca2+]i signalling in human spermatozoa induced by follicular fluid
Source: Hum Reprod. 2017 Aug 28;32(10):1995–2006. doi: 10.1093/humrep/dex269 (PMC5850303; doi:10.1093/humrep/dex269)
Supplement: Supplementary Data [file dex269_supplementaryfigures3.pdf]

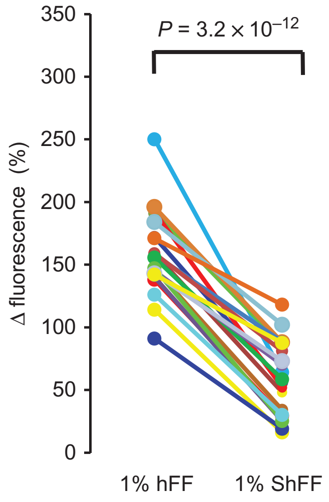

Supplementary Figure S3 Amplitude of $[Ca^{2+}]_i$ transient induced by 1% hFF before (left) and after (right) 'stripping' with dextran-coated charcoal. Data from 21 paired recordings using five different hFF samples are shown. $P = 3.2 \times 10^{-12}$ (paired *t*-test).
