# Supplementary material for: Complex CatSper-dependent and independent [Ca2+]i signalling in human spermatozoa induced by follicular fluid
Source: Hum Reprod. 2017 Aug 28;32(10):1995–2006. doi: 10.1093/humrep/dex269 (PMC5850303; doi:10.1093/humrep/dex269)
Supplement: Supplementary Data [file dex269_supplementaryfigures4.pdf]

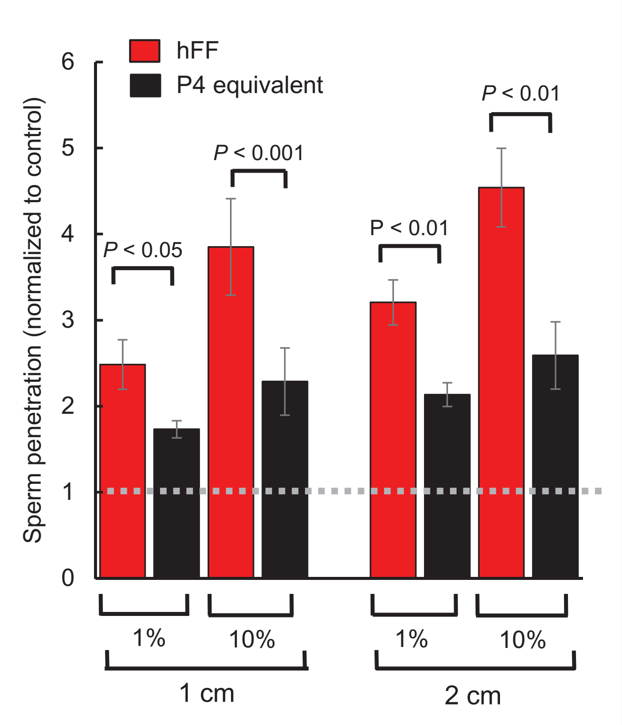

Supplementary Figure S4 Effects of exposure to hFF or equivalent P4 concentration on sperm penetration into viscous medium. Data for number of sperm penetrating to 1 cm (left panel) and 2 cm (right panel) have all been normalized to the parallel, untreated control (control response indicated by grey dashed line). All bars show mean ± SEM of six experiments using five different hFF. Red bars show hFF (1 and 10% as marked below *x*-axis), black bars show progesterone (P4) at equivalent dose to 1% hFF. All treatments were significantly different from control ($P < 0.005$). Statistical significance markers indicate comparison of effect of hFF with equivalent dose of P4.
