# Supplementary material for: Complex CatSper-dependent and independent [Ca2+]i signalling in human spermatozoa induced by follicular fluid
Source: Hum Reprod. 2017 Aug 28;32(10):1995–2006. doi: 10.1093/humrep/dex269 (PMC5850303; doi:10.1093/humrep/dex269)
Supplement: Supplementary Data [file dex269_supplementaryfigures5.pdf]

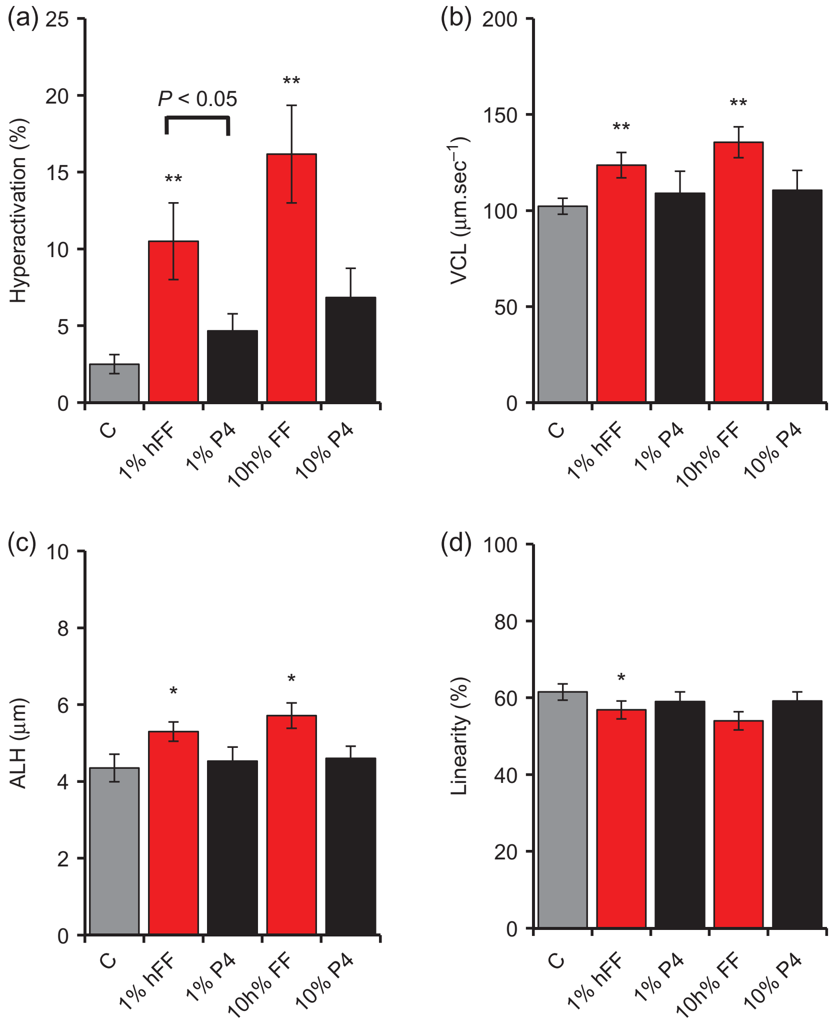

Supplementary Figure S5 Effects of exposure to hFF or equivalent progesterone concentration on sperm hyperactivation and key kinematic parameters assessed by CASA. Bars show mean ± SEM for (**a**) hyperactivation (%); (**b**) curvilinear velocity (VCL; µm.s^{-1}), (**c**) amplitude of lateral head movement (ALH; µm), (**d**) linearity (%). All bars show mean ± SEM of six experiments using five different hFF. Grey bars show control, red bars show hFF (1 and 10%), black bars show progesterone (P4) at equivalent dose to 1% hFF (1% P4) or 10% hFF (10% P4). Asterisks indicate significant difference from control (*$P < 0.05$; **$P < 0.01$). CASA, Computer-assisted sperm analysis.
