# Supplementary material for: Complex CatSper-dependent and independent [Ca2+]i signalling in human spermatozoa induced by follicular fluid
Source: Hum Reprod. 2017 Aug 28;32(10):1995–2006. doi: 10.1093/humrep/dex269 (PMC5850303; doi:10.1093/humrep/dex269)
Supplement: Supplementary Data [file dex269_supplementaryfiles1.pdf]

Human Reproduction, 2017
doi:10.1093/humrep/dex269

human reproduction SUPPLEMENTARY DATA

Supplementary File S1

A. Salines

HEPES buffered saline solution consisted of (in mM): $CaCl_2$, 1.8; KCl, 5.4; $MgSO_4 7 H_2O$, 0.8; NaCl, 116.4; NaH_2PO_4, 1; D-glucose, 5.5; sodium pyruvate, 2.73; sodium lactate, 41.75; HEPES, 25; BSA, 0.3% (w/v); pH adjusted to 7.4 using NaOH.

Bicarbonate buffered capacitating medium consisted of (in mM): $CaCl_2$, 1.8; KCl, 5.4; $MgSO_4 7 H_20$, 0.8; NaCl, 116.4; NaH_2PO_4 1; D-glucose, 5.5; sodium pyruvate, 2.73; sodium lactate, 41.75; sodium bicarbonate, 26; BSA, 0.3% (w/v); pH adjusted to 7.4 using NaOH.

Supplemented Earle's balanced salt solution (sEBSS) contained (in mM): NaH_2PO_4, 1.02; KCl, 5.4; $MgSO_4$, 0.811; D-glucose, 5.5; Na pyruvate, 2.5; Na lactate, 19.0; $CaCl_2$,1.8; $NaHCO_3$,,25.0; NaCl, 118.4 and HEPES, 15 (pH 7.4), supplemented with 0.3% (w/v) BSA.

Standard bath solution consisted of (in mM): NaCl, 135; KCl, 5; CaCl2, 2; MgSO4, 1; HEPES, 20; Glucose, 5; Na pyruvate, 1; Lactic acid, 10; pH adjusted to 7.4 with NaOH which brought [Na^+] to 154 mM.

Standard pipette solution consisted of (mM): NaCl, 10; KCl, 18; K gluconate, 92; MgCl2, 0.5, CaCl2, 0.6; EGTA, 1; HEPES, 10; pH adjusted to 7.4 using KOH which brought [K+] to 114 mM and [$Ca2^+$]i to 0.1 μM. [$Ca2^+$] in buffered solutions was calculated using MaxChelator (Maxchelator.stanford.edu).

Cs^+-based pipette solution contained Cs-methanesulphonate, 130 mM; HEPES, 40 mM; Tris-HCl, 1 mM; EGTA, 3 mM; EDTA, 3 mM, pH adjusted to 7.4 with CsOH.

Cs^+-based bath solution contained Cs-methanesulphonate, 140 mM; HEPES, 40 mM; EGTA, 3 mM; EDTA, 3 mM pH adjusted to 7.4 with CsOH.

CatSper tail current (Ba^{2+}) bath solution contained 10 mM $BaCl_2$, 140 mM NMDG, 100 mM HEPES, pH 7.4 with $HMeSO_3$.

CatSper tail current pipette solution contained 145 mM NMDG, 100 mM HEPES, 10 mM BAPTA, 0.5 mM Tris-HCl, pH 7.4 with $HMeSO_3$.

B. Dextran-coated charcoal solution

Dextran-coated charcoal was prepared by mixing 4 C charcoal (0.25% w/v) and dextran T-70 (0.0025% w/v) in a solution containing 1.5 mM $MgCl_2$, 10 mM HEPES and 0.25 M sucrose, pH 7.4 with NaOH and kept at 4°C. A volume of dextran-coated charcoal mixture double that of the volume of hFF to be steroid stripped was centrifuged to pellet the charcoal. The supernatant was removed and replaced with hFF. The charcoal was mixed with the hFF and incubated overnight at 4°C. To remove the charcoal, the hFF/charcoal mix was centrifuged at 1000 g for 5 min and the hFF was removed and filtered using a 0.22 μm filter. A paired sample of the same hFF not incubated with dextran-coated charcoal was also left overnight at 4°C (referred to as time-control).
